# Assessment of Nicotine and Cannabis Vaping and Respiratory Symptoms in Young Adults

**DOI:** 10.1001/jamanetworkopen.2020.30189

**Published:** 2020-12-22

**Authors:** Jessica L. Braymiller, Jessica L. Barrington-Trimis, Adam M. Leventhal, Talat Islam, Afton Kechter, Evan A. Krueger, Junhan Cho, Isabella Lanza, Jennifer B. Unger, Rob McConnell

**Affiliations:** 1 Department of Preventive Medicine, University of Southern California (USC), Los Angeles; 2 USC Norris Comprehensive Cancer Center, Keck Medicine of USC, Los Angeles; 3 Department of Psychology USC, Los Angeles; 4 Department of Human Development, California State University Long Beach, Long Beach

## Abstract

**Importance:**

Rates of e-cigarette use (ie, nicotine vaping) and cannabis vaping continue to increase among youth and young adults. However, the association of nicotine and cannabis vaping with independent respiratory health outcomes has not been well studied.

**Objective:**

To investigate associations of nicotine and cannabis vaping with bronchitic symptoms, wheeze, and shortness of breath.

**Design, Setting, and Participants:**

Cross-sectional survey data on self-reported lifetime, 6-month, and 30-day vaping from 2553 young adults recruited from high schools in Southern California were collected from June 2018 to October 2019. Of these participants, 94% provided data for shortness of breath and wheeze, and 86% provided data for chronic bronchitis.

**Main Outcomes and Measures:**

Self-reported nicotine and cannabis vaping were measured on a Likert-type scale using the following responses: (1) never used, (2) lifetime but no past 6-month use, (3) past 6-month use but no use in the past 30 days, (4) use on 1 or 2 of the past 30 days, and (5) use on 3 or more of the past 30 days. Three respiratory health outcomes were assessed separately on the basis of self-reported symptoms: bronchitic symptoms in the previous 12 months (ie, daily cough for 3 months in a row, congestion or phlegm other than with a cold, and/or bronchitis), wheeze in the previous 12 months, and shortness of breath when hurrying on level ground or walking up a slight hill.

**Results:**

Of 2553 participants in the analytic sample (mean [SD] age, 19.3 [0.79] years; 1477 [57.9%] female individuals), 1095 of 2553 young adults (42.9%) reported vaping nicotine and 939 of 2553 (38.4%) reported vaping cannabis. Compared with those who never vaped cannabis, individuals who vaped cannabis in their lifetime but not in the past 60 months (204 of 2553 [8.4%]; adjusted odds ratio [aOR], 1.83 [95% CI, 1.08-3.10]), in the past 6 months but not in the last 30 days (490 of 2443 [20.1%]; aOR, 1.58 [95% CI, 1.02-2.46]), 1-2 days in the past 30 days (90 of 2443 [3.7%]; aOR, 2.83 [95% CI, 1.46-5.50]), and 3 or more days in the past 30 days (155 of 2443 [6.3%]; aOR, 2.14 [95% CI, 1.16-3.92]) had significantly higher odds of chronic bronchitic symptoms after adjusting for nicotine vaping, cigarette smoking, cannabis smoking, and sociodemographic characteristics. Cannabis vaping 3 or more times in the last 30 days was also associated with increased odds of wheeze (aOR, 2.27 [95% CI, 1.17-4.37]). Associations of cannabis vaping with shortness of breath and nicotine vaping with any respiratory health outcome were not statistically significant in fully adjusted models.

**Conclusions and Relevance:**

Findings of this cross-sectional study suggest that cannabis vaping is associated with increased risk of bronchitic symptoms and wheeze in young adults. Further research is needed to understand the temporality of the association and the mechanisms underlying the difference between nicotine and cannabis vaping in the risk of bronchitic symptoms and wheeze.

## Key Points

**Question:**

Are nicotine and cannabis vaping associated with shortness of breath, wheeze, and bronchitic symptoms in young adults?

**Findings:**

In this cross-sectional study of 2553 young adults, cannabis vaping at any level was associated with increased odds of bronchitic symptoms, and cannabis vaping 3 or more times in the last month was associated with increased odds of wheeze, even after simultaneously adjusting for nicotine vaping, cigarette smoking, and combustible cannabis use.

**Meaning:**

Cannabis vaping may be associated with bronchitic symptoms in young adults, beyond the consequences of other drug use behaviors with shared risk pathways.

## Teaser

This cross-sectional study examines whether nicotine vaping and cannabis vaping are associated with bronchitic symptoms, wheeze, and shortness of breath in young adults.

## Introduction

The increasing prevalence of nicotine and cannabis vaping (ie, inhalation of aerosolized nicotine or cannabis containing e-liquids or oils) among youth and young adults is a serious public health concern.^1,2,3,4^ E-liquid aerosols contain many known respiratory toxicants, including volatile carbonyls (eg, aldehydes), furans, and metals,^5^ that likely adversely affect lung health with prolonged exposure.^6^ Because of the lack of regulation for the manufacture of nicotine and cannabis vaping devices, many e-liquids contain flavorings and other additives that may adversely affect respiratory health. For example, vitamin E acetate has been identified as an additive in tetrahydrocannabinol (THC)-containing e-liquids and played a role in the 2019 outbreak of e-cigarette and vaping-associated lung injury.^7^ This outbreak demonstrated that the health outcomes associated with vaping are not well understood and emphasized the urgent need to understand the implications of nicotine and cannabis vaping for respiratory health.

Experimental studies show that exposure to nicotine and cannabis containing e-liquid aerosols harm the respiratory system; however, human observational research on respiratory symptoms associated with vaping are limited.^6^ Some observational and epidemiologic studies have reported associations between nicotine vaping and chronic respiratory outcomes (eg, chronic cough, phlegm, bronchitis, asthma) among both adolescents and adults that are independent of combustible cigarette use.^6,8,9^ However, these studies relied on data collected prior to the emergence of a new class of vaping products (ie, pod mods such as JUUL [JUUL Labs, Inc]) that use a salt-based nicotine formulation and have other chemical constituents that differ from previously used nicotine vaping devices. It is also unclear if these associations are independent of cannabis vaping, a method of cannabis administration that has recently gained popularity among youth and young adults.^10^

Very little is known about the respiratory health outcomes associated with vaping liquid hash oil and other highly concentrated forms of THC, the psychoactive component of cannabis. Existing research on the respiratory health outcomes associated with cannabis has focused on combustible, smoked cannabis; several observational studies suggest that frequent combustible cannabis smoking is associated with increased odds of shortness of breath, wheeze, and bronchitic symptoms.^11,12,13,14,15^ Additional research is needed to assess the implications of other methods of cannabis administration, particularly cannabis vaping, for respiratory health. The present study examined associations between both nicotine and cannabis vaping and respiratory symptoms among young adults in Southern Californian from 2018 through 2019. Level of exposure to nicotine and cannabis vaping are examined in relation to (1) bronchitic symptoms, (2) wheeze, and (3) shortness of breath.

## Methods

### Analytic Sample

Data are from the Happiness and Health Study,^16^ a sample of young adults originally recruited from high schools throughout Southern California. Data for the current study were collected from June 2018 to October 2019, 1 year after participants graduated from high school. A total of 2553 participants provided complete data on lifetime, past 6-month, and past 30-day vaping (75% participation rate). Of these participants, 2389 (94%) provided complete data for shortness of breath, 2396 (94%) provided complete data for wheeze, and 2194 (86%) provided complete data for chronic bronchitis. Participants who did not provide any respiratory health data were less likely to report never vaping nicotine and smoking cigarettes (eTable 1 in the Supplement). This study was approved by the institutional review board of the University of Southern California. Written informed consent was obtained from all participants prior to data collection. This study followed the Strengthening of Reporting of Observational Studies in Epidemiology (STROBE) reporting guideline.

### Measures

#### Level of Nicotine and Cannabis Vaping

Participants responded to the question, “Have you ever used the follow substances in your life?” Specific substances included any e-cigarette with nicotine, JUUL or similar device, another electronic vaping device, or an electronic device to vape THC or hash oil/dabs (eg, wax, shatter, budder, and butane hash oil), cigarettes, smoking marijuana, and blunts (questionnaire is available in the eAppendix in the Supplement). Response options included no, yes, yes but not in the last 6 months, or yes in the last 6 months. The term *dabs* refers to vaping concentrated cannabis.^17,18^ For each question, response options were no, yes but not in the last 6 months, or yes in the last 6 months. Participants who reported using the substance within the last 6 months were then asked to indicate how many total days within the past month they used that substance. Response options included 0 days, 1 or 2 days, 3 to 5 days, 6 to 9 days, 10 to 19 days, 20 to 29 days, and all 30 days. Participants who responded “no” or “yes but not in the last 6 months” to the first question did not receive questions about use in the last month.

Information from each of the questions was combined to create two 5-level categorical variables, 1 for nicotine vaping and another for cannabis vaping. Level of nicotine vaping was determined based on responses for items on the use of e-cigarettes with nicotine, JUUL or similar devices, and other electronic vaping devices. Level of cannabis vaping was determined based on responses for items regarding the use of electronic devices to vape THC or hash oil, or vaping concentrated cannabis/dabs. Categorical response options were coded as (1) never used, (2) used in the lifetime but not in the past 6 months, (3) used in the past 6 months but not in the past 30 days, (4) used 1 or 2 days in the past 30 days, and (5) used 3 or more days in the past 30 days.

#### Respiratory Outcomes

Based on previous reports of respiratory symptoms among individuals who vape nicotine,^6,9^ we assessed 3 different respiratory outcomes: bronchitic symptoms, wheeze, and shortness of breath. Given the heterogeneity in the pathology of these respiratory symptoms, each outcome was examined separately. Bronchitic symptoms represent a clinical condition reflecting irritant exposure, wheeze is characteristic of bronchoconstriction, and shortness of breath is nonspecific but may reflect bronchiolitis obliterans, which has been associated with the diketones present in e-liquids.^19^ Wheeze was chosen as a main respiratory outcome over an asthma diagnosis because most asthma has onset in early life, before the initiation of vaping or smoking. Therefore, wheeze is considered to be a better reflection of current respiratory health outcomes.

To assess bronchitic symptoms, participants reported whether they had (1) daily cough in the morning for 3 months in a row, (2) daily cough at other times of the day for 3 months in a row, (3) congestion or phlegm other than when accompanied by a cold, and (4) bronchitis in the past 12 months. Participants were considered to have bronchitic symptoms if they reported having any of the 4 symptoms.^6,9^ For wheeze, participants reported whether they had experienced wheezing or whistling in the chest during the previous 12 months (yes or no). For shortness of breath, participants reported whether they had been troubled by shortness of breath when hurrying on level ground or walking up a slight hill (yes or no).

#### Covariates

Sociodemographic characteristics, as well as level of cigarette and combustible cannabis smoking were included in the analysis as covariates (eAppendix in the Supplement). Questionnaires assessed race/ethnicity (Hispanic, non-Hispanic [NH] white, NH Asian, NH African American, and NH other categories [ie, Pacific Islander, Native American/Alaska Native, multiracial]), sex, and height and weight (converted into body mass index [BMI; calculated as weight in kilograms divided by height in meters squared]). To assess current personal financial status, participants were asked, “Considering your own income and the income from any other people who help you, how would you describe your overall personal financial situation?” Response options included: “live comfortably,” “meet needs with a little left,” “just meet basic expenses,” and “do not meet basic expenses.”

Level of cigarette smoking and combustible cannabis use (ie, marijuana cigarettes, joints, blunts) were assessed and recoded in the same way as nicotine and cannabis vaping. Two separate 5-level variables reflected never use (0), used in the lifetime but not in the past 6 months (1), used in the past 6 months but not in the past 30 days (2), used 1 or 2 days in the past 30 days (3), used 3 or more days in the past 30 days (4).

### Statistical Analyses

A series of logistic regression models were used to examine the associations between the level of nicotine and cannabis vaping with shortness of breath, wheeze, and symptoms of bronchitis in separate models. First, models were adjusted for sociodemographic characteristics including age, sex, race/ethnicity, personal financial status, and BMI. Next, models that were simultaneously adjusting for both nicotine and cannabis vaping examined associations of vaping with bronchitic symptoms, wheeze, and shortness of breath. These models additionally adjusted for cigarette smoking and combustible cannabis use in order to further assess the independent implications of each substance and the method of administration. Two-sided *P* < .05 was considered to be statistically significant. Results are presented as adjusted odds ratios (aORs) and 95% confidence intervals. Benjamini-Hochberg adjusted *P* values were used to correct for multiple 2-tailed tests. Analyses were performed using SAS software, version 9.4 (SAS Institute, Inc).

## Results

Among the 2553 participants included in the analytic sample, 1477 were female (57.9%), with a mean (SD) age of 19.3 (0.79) years (Table 1). Most young adults were Hispanic (1188 [47.3%]), with the remainder of the sample composed of individuals who were non-Hispanic (NH) Asian (454 [18.1%]), NH white (413 [16.5%]), NH Multiracial (166 [6.6%]), NH Black (120 [4.8%]), or of another race/ethnicity (43 [1.7%] did not indicate their race/ethnicity or had missing data). Mean (SD) BMI for young adults in the analytic sample was 24.97 (6.51). Of 2392 participants, 1081 (45.2%) reported living comfortably; 161 (6.3%) did not indicate their current financial status. Most participants reported never smoking cigarettes (1960 [76.9%]) or combustible cannabis (1307 [51.3%]).

**Table 1.** Descriptive Characteristics of the Analytic Sample^a^

Covariates	No. (%)
Total participants	2553
Sex	
Female	1477 (57.9)
Male	1076 (42.1)
Race/ethnicity^b^	
Hispanic	1188 (47.3)
Non-Hispanic	
Asian	454 (18.1)
White	413 (16.5)
Multiracial	166 (6.6)
Black	120 (4.8)
Native Hawaiian or Pacific Islander	109 (4.3)
Another racial/ethnic category	60 (2.4)
Personal financial status	
Live comfortably	1081 (45.2)
Meets needs with little left	678 (28.3)
Just meets basic expenses	506 (21.2)
Do not meet basic expenses	127 (5.3)
Age, mean (SD), y	19.3 (0.79)
BMI, mean (SD)^c^	24.97 (6.51)
Frequency of combustible cigarette use	
Never used	1960 (76.9)
Lifetime but not in the past 6 mo	269 (10.6)
Past 6 mo but not in the past 30 d	102 (4.0)
1-2 d In the past 30 d	121 (4.8)
≥3 d In the past 30 d	98 (3.8)
Frequency of combustible cannabis use	
Never used	1307 (51.3)
Lifetime, but not in the past 6 mo	323 (12.73)
Past 6 mo, but not in the past 30 d	182 (7.1)
1-2 d In the past 30 d	219 (8.6)
≥3 d In the past 30 d	517 (20.3)

Abbreviation: BMI, body mass index.

^a^ Frequency and sample size reported unless otherwise noted.

^b^ Forty-three individuals [1.7%] did not indicate their race/ethnicity or had missing data.

^c^ Body Mass Index scores (calculated as weight in kilograms divided by height in meters squared): 18.5 to 24.9 indicates normal or healthy weight; 25.0 to 29.9 indicates overweight.

### Respiratory Health Outcomes and Levels of Nicotine and Cannabis Vaping

Overall, 384 of 2194 individuals (17.5%) in the analytic sample reported bronchitic symptoms, 312 of 2396 (13.0%) reported wheeze in the past year, and 488 of 2389 (20.4%) reported shortness of breath (Table 2).

**Table 2.** Unadjusted Frequency of Self-reported Bronchitic Symptoms, Wheeze, and Shortness of Breath, by Each Exposure Variable

Exposures	No./Total (%)	Prevalence of each respiratory outcome, No./Total (%)^**a**^		
		Bronchitic symptoms^b^	Wheeze^c^	Shortness of breath^d^
Total	2553	384/2194 (17.5)	312/2396 (13.0)	488/2389 (20.4)
**Frequency of nicotine vaping**^e^				
Never used	1456/2553 (57.1)	191/384 (49.7)	160/312 (51.3)	250/488 (51.2)
Lifetime but not in the past 6 mo	268/2553 (10.5)	43/384 (11.2)	31/312 (9.9)	51/488 (10.5)
Past 6 mo but not in the past 30 d	228/2553 (8.9)	35/384 (9.1)	37/312 (11.9)	55/488 (11.3)
1-2 d In the past 30 d	199/2553 (7.8)	36/384 (9.4)	26/312 (8.3)	50/488 (10.3)
≥3 d In the past 30 d	400/2553 (15.7)	79/384 (20.6)	58/312 (18.6)	82/488 (16.8)
**Frequency of cannabis vaping**^f^				
Never used	1504/2553 (58.9)	185/384 (48.2)	155/312 (49.7)	256/488 (52.5)
Lifetime but not in the past 6 mo	204/2553 (8.0)	35/384 (9.1)	23/312 (7.4)	34/488 (7.0)
Past 6 mo but not in the past 30 d	490/2553 (19.2)	82/384 (21.4)	72/312 (23.1)	117/488 (24.0)
1-2 d In the past 30 d	90/2553 (3.5)	24/384 (6.3)	15/312 (4.8)	21/488 (4.3)
≥3 d In the past 30 d	155/2553 (6.1)	36/384 (9.4)	31/312 (9.9)	39/488 (8.0)

^a^ Column percentages for yes response reported for each respiratory health symptom.

^b^ Data for 359 participants was missing or not provided.

^c^ Data for 157 participants was missing or not provided.

^d^ Data for 164 participants was missing or not provided.

^e^ No data on nicotine vaping (n = 2).

^f^ No data on cannabis vaping (n = 110).

In the analytic sample, 1456 of 2553 young adults (57.1%) reported never vaping nicotine (Table 2), 268 (10.5%) reported vaping nicotine in their lifetime but not within the past 6 months, and 228 (8.9%) reported use within the past 6 months but not the past 30 days. A total of 199 young adults (7.8%) vaped nicotine 1 to 2 days in the past 30 days, and 400 (15.7%) vaped 3 or more days in the past 30 days. Two individuals did not provide data on nicotine vaping.

In the analytic sample, 1504 of 2553 young adults (61.6%) reported never vaping cannabis and 939 (38.4%) reported vaping cannabis in their lifetime (Table 2). A total of 204 (8.4%) reported vaping cannabis in their lifetime but not within the past 6 months, 490 (20.1%) reported use within the past 6 months but not the past 30 days, 90 (3.7%) vaped cannabis 1 to 2 days in the past 30 days, and 155 (6.3%) vaped 3 or more days in the past 30 days (110 individuals did not provide data on cannabis vaping).

### Associations of Nicotine and Cannabis Vaping With Respiratory Health Symptoms

Details on the associations of nicotine and cannabis vaping with bronchitic symptoms, wheeze, and shortness of breath are presented in Table 3.

**Table 3.** Associations Between Frequency of Vaping Nicotine and Cannabis With Bronchitic Symptoms, Wheeze, and Shortness of Breath

	aOR (95% CI)					
	Bronchitic symptoms (n = 2194)		Wheeze (n = 2396)		Shortness of breath (n = 2389)	
	Sociodemographic adjustment^a^	Full adjustment^b^	Sociodemographic adjustment	Full adjustment	Sociodemographic adjustment	Full adjustment
**Frequency of vaping nicotine**						
Never used	1 Reference	1 Reference	1 Reference	1 Reference	1 Reference	1 Reference
Lifetime but not in the past 6 mo	1.35 (0.92-1.97)	1.21 (0.76-1.93)	1.13 (0.74-1.71)	0.96 (0.58-1.59)	1.2 (0.85-1.71)	1.03 (0.68-1.57)
Past 6 mo but not in the past 30 d	1.18 (0.79-1.78)	0.82 (0.50-1.32)	1.55 (1.04-2.32)	1.06 (0.66-1.71)	1.41 (0.99-2.00)	1.10 (0.73-1.67)
1-2 d in the past 30 d	1.48 (0.97-2.21)	1.13 (0.71-1.81)	1.27 (0.81-1.99)	0.99 (0.59-1.65)	1.64 (1.14-2.36)	1.31 (0.86-1.99)
≥3 d in the past 30 d	1.78 (1.32-2.41)	0.96 (0.63-1.46)	1.41 (1.01-1.96)	0.85 (0.54-1.35)	1.45 (1.08-1.96)	0.96 (0.64-1.42)
**Frequency of vaping cannabis**						
Never used	1 Reference	1 Reference	1 Reference	1 Reference	1 Reference	1 Reference
Lifetime but not in the past 6 mo	1.47 (0.97-2.23)	1.73 (1.01-2.96)	1.06 (0.65-1.73)	1.12 (0.61-2.03)	0.95 (0.63-1.44)	0.90 (0.57-1.40)
Past 6 mo but not in the past 30 d	1.44 (1.07-1.94)	1.63 (1.04-2.56)	1.47 (1.08-2.00)	1.47 (0.92-2.35)	1.35 (1.04-1.75)	1.25 (0.86-1.84)
1-2 d in the past 30 d	2.95 (1.75-4.98)	2.79 (1.42-5.47)	1.76 (0.96-3.13)	1.84 (0.88-3.86)	1.67 (0.98-2.86)	1.55 (0.81-2.96)
≥3 d in the past 30 d	2.51 (1.63-3.84)	2.19 (1.18-4.06)	2.13 (1.35-3.36)	2.27 (1.17-4.37)	1.67 (1.09-2.53)	1.41 (0.80-2.48)

Abbreviation: aOR, adjusted odds ratio.

^a^ Associations of nicotine and cannabis vaping with bronchitic symptoms, wheeze, and shortness of breath, adjusting for sociodemographic factors including sex, age, race/ethnicity, personal financial status, and body mass index (calculated as weight in kilograms divided by height in meters squared). Each respiratory health outcome was modeled separately by exposure. All available data were used in each model; samples vary between models due to missing data in the outcome variables.

^b^ Associations of nicotine and cannabis vaping with bronchitic symptoms, wheeze, and shortness of breath, simultaneously adjusted for vaping nicotine and vaping cannabis, as well as sociodemographic characteristics and frequency of combustible cigarette and combustible cannabis use. Each respiratory health outcome was modeled separately. All available data were used in each model; samples vary between models due to missing data in the outcome variables.

When adjusting for sociodemographic characteristics only, vaping nicotine 3 days or more in the past 30 days was associated with increased odds of bronchitic symptoms (aOR, 1.78; 95% CI, 1.32-2.41), as was vaping cannabis in the past 6 months but not the past 30 days (aOR, 1.44; 95% CI, 1.07-1.94), 1 to 2 days in the past 30 days (aOR, 2.95; 95% CI, 1.75-4.98), and 3 or more days in the past 30 days (aOR, 2.51; 95% CI, 1.63-3.84). After fully adjusting for vaping cannabis, combustible cigarette, and cannabis smoking, vaping cannabis was associated with bronchitic symptoms at every level of use (aORs range from 1.63-2.79), with a stronger association observed for use on 1 to 2 days in the past 30 days (aOR, 2.79; 95% CI, 1.42-5.47) and 3 or more days in the past 30 days (aOR, 2.19; 95% CI, 1.18, 4.06). There were no statistically significant associations between vaping nicotine and bronchitic symptoms in the fully adjusted model (aORs range from 1.63-2.79 [95% CI, 1.01-5.47]).

When adjusting for sociodemographic characteristics only, vaping nicotine in the past 6 months but not in the past 30 days (aOR, 1.55; 95% CI, 1.04-2.32) and 3 or more days in the past 30 days (aOR, 1.41; 95% CI, 1.01-1.96) was associated with increased odds of wheeze, as was vaping cannabis in the past 6 months but not in the past 30 days (aOR, 1.47; 95% CI, 1.08-2.00) and 3 or more days in the past 30 days (aOR, 2.13; 95% CI, 1.35-3.36). After full adjustment, vaping cannabis 3 or more days in the past 30 days was associated with increased odds of wheeze (aOR, 2.27; 95% CI, 1.17-4.37).There were no statistically significant associations between vaping nicotine and wheeze in the fully adjusted model (aORs range from 0.96-1.06).

When adjusting for sociodemographic characteristics only, vaping nicotine 1 to 2 days in the past 30 days (aOR, 1.64; 95% CI, 1.14-2.36) and 3 or more days in the past 30 days (aOR, 1.45; 95% CI, 1.08-1.96) was associated with shortness of breath. After sociodemographic adjustment, vaping cannabis in the past 6 months but not in the past 30 days (aOR, 1.35; 95% CI, 1.04-1.75), 1 to 2 days (aOR, 1.67; 95% CI, 0.98-2.86), and 3 or more days in the past 30 days (aOR, 1.67; 95% CI, 1.09-2.53) was also associated with shortness of breath. After full adjustment, there were no statistically significant associations between vaping nicotine (aORs range from 0.96-1.31) or vaping cannabis (aORs range from 0.90-1.55) with shortness of breath.

## Discussion

This study provides new evidence for an independent association between any level of cannabis vaping and persistent symptoms of bronchitis (daily cough, congestion, or phlegm, and/or a diagnosis of bronchitis in the past 12 months). Frequency of cannabis vaping was associated with increased odds of bronchitic symptoms at all levels of use when compared with those who never vaped cannabis, even after simultaneously adjusting for nicotine vaping, sociodemographic characteristics, cigarette smoking, and combustible cannabis use. Effect size estimates were 2- to 3-fold larger among participants who vaped cannabis in the past 30 days compared with those who never vaped cannabis. In sociodemographic adjusted models, vaping nicotine on 3 or more days in the past 30 days was associated with increased odds of bronchitic symptoms and wheeze, and vaping nicotine on 1 or more days in the past 30 days was associated with increased odds of shortness of breath. However, these associations were confounded by smoking combustible cigarettes and vaping or smoking cannabis in the fully adjusted models. Cannabis vaping on 3 or more days in the past 30 days was also associated with increased odds of wheezing or whistling in the chest during the past 12 months. Compared with those who never vaped cannabis, the fully adjusted model showed that odds of reporting wheeze increased more than 2-fold among those who vaped cannabis 3 or more times in the past 30 days.

Statistically significant associations of vaping nicotine with wheeze and shortness of breath were observed in models that were adjusted only for sociodemographic characteristics; however, the results were not statistically significant in fully adjusted models. Residual confounding with vaping cannabis and/or smoking cigarettes or combustible cannabis could partially explain these results. Nicotine vaping among young people has been linked to increased risk for combustible cigarette smoking,^20,21^ cannabis use,^22,23^ and other substance use behaviors,^24^ and there is considerable overlap in these substance use behaviors in the analytical sample (eTable 2 in the Supplement). However, models simultaneously adjusting for both nicotine and cannabis vaping as well as smoking cigarettes and combustible cannabis performed well, allowing us to separate the outcomes associated with use of each substance and method of administration. Future research in populations of exclusive nicotine vapers and exclusive cannabis vapers are needed. Prospective studies are also needed to specify temporal associations.

Although the exact mechanisms underlying associations of cannabis vaping with respiratory health are unclear and warrant further study, there are several factors that could contribute to the increased risk of bronchitic symptoms. Vitamin E acetate in THC-containing e-liquids has been associated with vaping-related lung injury^7,25,26,27^; however, this kind of vaping has virtually disappeared, and was largely associated with illicit products that are not widely used. Other distinct and important differences in the chemical constituents of THC-containing vs nicotine-containing e-liquids persist and may be contributing to respiratory symptoms in those who vape cannabis. Product characteristics (eg, temperature of heating element, device type) may also be different. Because most young people obtain cannabis vaping devices from their peers, dealers, and/or online,^7^ there is increased potential for customizing or tampering with THC-containing e-liquids. The large number of available vaping devices (eg, over 150 different brands of THC-containing and nicotine-containing vaping products^7^) coupled with the lack of regulation of these devices present unique challenges to characterizing chemical constituents of e-liquids and the associated adverse health outcomes.

Our measure of cannabis vaping included dabbing. Dabs are highly concentrated forms of cannabis oil that are made by extracting THC and other cannabinoids from the cannabis plant using butane; the resulting substance is a sticky, waxy oil. Inhaling aerosolized forms of highly concentrated dabs and/or THC or hash oils may confer increased risk for acute and chronic respiratory symptoms. Another factor contributing to differences in risk may be variations in vaping topography (eg, number of puffs, puff volume, and duration) for cannabis-vaping vs nicotine-vaping. For example, behavioral studies show that both tobacco and cannabis smokers can adjust their smoking behaviors to increase yield and the psychoactive effects of the drug.^28,29^ Evidence suggests that vaping topography is also highly variable among those who vape nicotine; however, less is known about cannabis vaping. Based on data regarding combustible cannabis smoking behaviors and typography, we might infer that individuals who vape cannabis or vape cannabis concentrates may also adjust their vaping behavior, taking deeper and longer hits. Such differences may result in a higher dose or overall exposure to THC-containing aerosol, even at a lower frequency and intensity of use.

### Limitations

This study has limitations. The original sample was from a Southern California school population; 75% of the original sample participated in the current study and selection bias could be present. Although ethnically diverse, the sample is also not nationally representative, and findings may not be generalizable to other regions or to individuals who did not attend or complete high school. The current study was also cross-sectional; longitudinal research would help to elucidate a causal association between vaping and chronic respiratory symptoms in the United States over time. Research is needed to determine if the findings of the present study can be replicated longitudinally in large nationally representative data sets, such as the Population Assessment of Tobacco and Health Study. Although existing longitudinal research has reported associations of former and current nicotine vaping with obstructive pulmonary disease, chronic bronchitis, emphysema, or asthma in the Population Assessment of Tobacco and Health study,^30^ longitudinal research on the associations of cannabis vaping with respiratory symptoms, both independently and in the context of nicotine vaping, is lacking.

## Conclusions

In this cross-sectional study of young adults, vaping cannabis at any frequency was associated with increased risk of bronichitic symptoms, and vaping cannabis on 3 or more days in the past 30 days was associated with increased risk of wheeze. Results were independent of nicotine vaping and smoking combustible products. Vaping is a relatively new form of administering both nicotine and cannabis; the long-term health implications of use at the population level may not be observed for decades. Further research is needed to understand the temporality of the association and the mechanisms underlying the difference between nicotine and cannabis in risk of respiratory symptoms. In the meantime, nicotine and cannabis vaping warrant consideration in patient care and the development of public health policy.

## Notes

Supplement. **eTable 1.** Sample characteristics for participants in the analytic sample compared to non-participants **eTable 2.** Co-occurrence of nicotine vaping, cannabis vaping, cigarette smoking, and combustible cannabis use in the past 6 months **eAppendix.**
