# Supplementary material for: Assessment of Nicotine and Cannabis Vaping and Respiratory Symptoms in Young Adults
Source: JAMA Netw Open. 2020 Dec 22;3(12):e2030189. doi: 10.1001/jamanetworkopen.2020.30189 (PMC7756238; doi:10.1001/jamanetworkopen.2020.30189)
Supplement: Supplement. — eTable 1. Sample characteristics for participants in the analytic sample compared to non-participants eTable 2. Co-occurrence of nicotine vaping, cannabis vaping, cigarette smoking, and combustible cannabis use in the past 6 months eAppendix. [file jamanetwopen-e2030189-s001.pdf]

Supplemental Online Content

Braymiller JL, Barrington-Trimis JL, Leventhal AM, et al. Assessment of nicotine and cannabis vaping and respiratory symptoms in young adults. *JAMA Netw Open*. 2020;3(12):e2030189.
doi:10.1001/jamanetworkopen.2020.30189

eTable 1. Sample characteristics for participants in the analytic sample compared to non-participants
eTable 2. Co-occurrence of nicotine vaping, cannabis vaping, cigarette smoking, and combustible cannabis use in the past 6 months
eAppendix.

This supplemental material has been provided by the authors to give readers additional information about their work.

© 2020 Braymiller JL et al. *JAMA Network Open.*

**eTable 1.** Sample characteristics for participants in the analytic sample compared to non-participants					
	**Analytic Sample N=2,553**	**Completed Wave 9, but NO resp. data N=148**	**p-value**	**Did NOT Complete Wave 9 N= 654**	**p-value**
**Sex**			**0.00**		**<.0001**
Female	57.9%	46.0%		39.9%	
Male	42.2%	54.1%		60.1%	
**Race/Ethnicity**			0.61		**0.01**
Hispanic	47.3%	48.3%		49.3%	
NH Asian	18.1%	12.6%		12.1%	
NH White	16.5%	14.0%		15.8%	
NH Multiracial	6.6%	5.6%		6.9%	
NH Black	4.8%	5.6%		5.7%	
NH Native Hawaiian or Pacific Islander	4.3%	4.9%		3.2%	
NH Another racial/ethnic category	2.4%	5.6%		5.7%	
**Nicotine Vaping at Wave 9**			**0.01**		
Never used	57.1%	45.2%		--	
Lifetime, but not past 6 months	10.5%	19.9%		--	
Past 6 months, but not P30D	8.9%	8.9%		--	
1-2 days in the P30D	7.8%	6.2%		--	
3+ days in the P30D	15.7%	19.9%		--	
**Cannabis Vaping at Wave 9**			0.13	--	
Never used	61.6%	55.6%		--	
Lifetime, but not past 6 months	8.4%	13.3%		--	
Past 6 months, but not P30D	20.1%	17.8%		--	
1-2 days in the P30D	3.7%	4.4%		--	
3+ days in the P30D	6.3%	8.9%		--	
**Combustible Cigarette Use at Wave 9**			**0.01**	--	
Never used	76.9%	63.0%		--	
Lifetime, but not past 6 months	10.6%	17.1%		--	
Past 6 months, but not P30D	4.0%	4.1%		--	
1-2 days in the P30D	4.8%	8.9%		--	
3+ days in the P30D	3.8%	6.9%		--	
**Combustible Cannabis Use**			0.40	--	
Never used	51.3%	44.8%		--	
Lifetime, but not past 6 months	12.7%	16.6%		--	
Past 6 months, but not P30D	7.1%	6.2%		--	
1-2 days in the P30D	8.6%	9.0%		--	
3+ days in the P30D	20.3%	23.5%		--	

© 2020 Braymiller JL et al. *JAMA Network Open.*

eTable 2. Co-occurrence of nicotine vaping, cannabis vaping, cigarette smoking, and combustible cannabis use in the past 6 months.

	**Nicotine Vaping**	**Cannabis Vaping**	**Cigarette Smoking**	**Combustible Cannabis Use**
**Nicotine Vaping**	--	--	--	--
**Cannabis Vaping**	510 (67.4%)	--	--	--
**Cigarette Smoking**	266 (32.2%)	197 (26.8%)	--	--
**Combustible Cannabis Use**	588 (71.2%)	645 (88.0%)	250 (77.9%)	--

Note. Frequencies and column percentages for a 'yes' response are reported for each substance use behavior.

© 2020 Braymiller JL et al. *JAMA Network Open.*

eAppendix
QUESTIONNAIRE

Respiratory Outcomes.
Bronchitic Symptoms
1. During the last 12 months, have you had a cough first thing in the morning, that lasted for as much as 3 months in a row?

❑ No
❑ Yes
❑ Don't know

2. During the last 12 months, have you had a cough at any other times of the day that lasted for as much as 3 months in a row?

❑ No
❑ Yes
❑ Don't know

3. Other than with colds, do you usually seem congested in the chest or bring up phlegm?

❑ No
❑ Yes
❑ Don't know

4. During the past 12 months, have you had bronchitis?

❑ No
❑ Yes
❑ Don't know

Wheeze
Have you had wheezing or whistling in the chest in the last 12 months?

❑ No
❑ Yes

Shortness of Breath
Are you troubled by shortness of breath when hurrying on level ground or walking up a slight hill?

❑ No
❑ Yes

© 2020 Braymiller JL et al. *JAMA Network Open.*

Substance Use Information.

1. Have you ever used the following substances in your life?

	No	Yes, but not in the last 6 months	Yes, in the last 6 months
Any electronic cigarette **with nicotine** *(e-cigs, personal vaporizer, PV)*	❑	❑	❑
Juul or similar device	❑	❑	❑
Other electronic vaping device	❑	❑	❑
Electronic device to vape **THC or hash oil** *(liquid pot, cannabis oil, weed pen, PAX Era)*	❑	❑	❑
Dabbing marijuana concentrates *(Wax, shatter, budder, butane hash oil, BHO, concentrates)*	❑	❑	❑
Cigarettes *(Marlboro, Camel, Newport, etc.)*	❑	❑	❑
Smoking marijuana *(joints, marijuana cigarettes)*	❑	❑	❑
Blunts *(marijuana rolled in tobacco leaf or cigar casing)*	❑	❑	❑

2. In the last 30 days, how many total days have you used…?

	0 days	1 or 2 days	3 to 5 days	6 to 9 days	10 to 19 days	20-29 days	All 30 days
Any electronic cigarette **with nicotine** *(e-cigs, personal vaporizer, PV)*	❑	❑	❑	❑	❑	❑	❑
Juul or similar device	❑	❑	❑	❑	❑	❑	❑
Other electronic vaping device	❑	❑	❑	❑	❑	❑	❑
Electronic device to vape **THC or hash oil** *(liquid pot, cannabis oil, weed pen, PAX Era)*	❑	❑	❑	❑	❑	❑	❑
Dabbing marijuana concentrates *(Wax, shatter, budder, butane hash oil, BHO, concentrates)*	❑	❑	❑	❑	❑	❑	❑
Cigarettes *(Marlboro, Camel, Newport, etc.)*	❑	❑	❑	❑	❑	❑	❑
Smoking marijuana *(joints, marijuana cigarettes)*	❑	❑	❑	❑	❑	❑	❑
Blunts *(marijuana rolled in tobacco leaf or cigar casing)*	❑	❑	❑	❑	❑	❑	❑

© 2020 Braymiller JL et al. *JAMA Network Open.*

Demographics.

What is your sex assigned at birth?

❑ Male

❑ Female

Are you Hispanic or Latino?

❑ Yes

❑ No

Which of the following races do you identify with? (Check all that apply)

❑ American Indian or Alaska Native

❑ Asian

❑ Black or African American

❑ Native Hawaiian or Pacific Islander

❑ White

❑ Multi-ethnic or Multi-racial

❑ Other (please specify)

Please specify the race(s) with which you identify: ______________________

Considering your own income and the income from any other people who help you, how would you describe your overall personal financial situation?

❑ Live comfortably

❑ Meet needs with a little left

❑ Just meet basic expenses

❑ Don't meet basic expenses

How tall are you?

Feet _____________

Inches _____________

How much do you weigh?

_____________ pounds

© 2020 Braymiller JL et al. *JAMA Network Open.*
